# Gastrointestinal cancer-associated fibroblasts expressing Junctional Adhesion Molecule-A are amenable to infection by oncolytic reovirus

**DOI:** 10.1038/s41417-022-00507-9

**Published:** 2022-07-22

**Authors:** Tom J. Harryvan, Matteo Golo, Nicole Dam, Mark J. A. Schoonderwoerd, Elham Aida Farshadi, Marten Hornsveld, Rob C. Hoeben, Lukas J. A. C. Hawinkels, Vera Kemp

**Affiliations:** 1 Department of Gastroenterology & Hepatology, Leiden University Medical Center, Leiden, The Netherlands; 2 Department of Cell & Chemical Biology, Leiden University Medical Center, Leiden, The Netherlands; 3 Department of Pulmonary Medicine, Erasmus University Medical Center, Rotterdam, The Netherlands

**Keywords:** Gastrointestinal cancer, Cancer microenvironment, Tumour virus infections

## Abstract

Gastrointestinal (GI) cancers are characterized by extensive tumor stroma that both promotes tumor progression and acts as a physical barrier for adjacent tumor cells, limiting the effect of current treatment modalities. Oncolytic virotherapy is currently investigated in clinical trials as a novel therapeutic agent for different malignancies of the GI tract, but it is largely unknown whether these viruses can also target the tumor stroma. Here, we investigated the tropism of two commonly studied OVs, adenovirus and reovirus, towards primary GI fibroblasts from human oesophageal, gastric, duodenal and pancreatic carcinomas (*N* = 36). GI fibroblasts were susceptible to type 3 Dearing (T3D) strain R124 and bioselected mutant reovirus (*jin*-3) infection but not oncolytic adenovirus (Ad5-Δ24). Efficient infection and apoptosis of human and mouse GI cancer-derived fibroblasts by these reoviruses was partially dependent on the expression of the reovirus entry receptor, Junctional Adhesion Molecule-A (JAM-A). Moreover, human GI cancer organoid-fibroblast co-cultures showed higher overall infectivity when containing JAM-A expressing fibroblasts as compared to JAM-A negative fibroblasts, indicating a potential role of JAM-A expressing fibroblasts for viral dissemination. We further show that JAM-A is not only necessary for efficient reovirus infection of fibroblasts but also partially mediates reovirus-induced apoptosis, dependent on signaling through the C-terminal PDZ-domain of JAM-A. Altogether, our data show the presence of JAM-A expressing fibroblasts in both human and murine GI cancers that are amenable to infection and induction of apoptosis by reovirus, extending the potential anti-cancer actions of reovirus with stromal targeting.

## Introduction

Oncolytic virotherapy is currently investigated as a novel therapy for malignancies of the gastrointestinal (GI) tract [1, 2]. Various oncolytic viruses (OVs) are under investigation, of which adenoviruses and respiratory enteric orphan viruses (reoviruses) are amongst the most frequently tested [1]. The mode of action of these OVs is thought to be through a dual mechanism reliant on both selective killing of tumor cells as well as the induction of anti-tumor immunity [3]. A variety of wildtype, bioselected and genetically modified OVs exist that either have natural or acquired tumor tropism or enhanced immunogenic properties [4, 5]. Current selection and generation of suitable OVs is mostly tailored towards their ability to infect and kill epithelial cancer cells. However, the tumor microenvironment (TME), which is comprised of a multitude of cell types makes up a significant part of the tumor mass. Cells in the TME (in-)directly influence tumor progression and metastasis and their abundance is related to patient survival. Currently it is largely unknown whether cells in the TME are susceptible to OV targeting and what their impact is on the therapeutic success of oncolytic virotherapy.

As the main cellular constituent of the TME in GI malignancies, cancer-associated fibroblasts (CAFs) are involved in multiple aspects of tumor progression, including chemoresistance, immune evasion and tumor invasion [6]. Desmoplasia, the growth of fibrous tissue around the tumor cells, is a phenomenon observed in the majority of GI cancers [7] and a direct result of fibroblast proliferation and activation. This is thought to be involved in protecting the underlying tumor cells from therapies and anti-tumor immune responses. OV-mediated targeting of the tumor stroma therefore seems an attractive approach, in which the potential benefits are (1) disruption of the desmoplastic stromal barrier, enabling immune cell invasion and increased influx of therapeutics and (2) serve as a conduit for OV replication and subsequent infection of desmoplastic-adjacent tumor cells. Several genetically modified OVs have been generated with engineered CAF tropism [8–12] through transgene incorporation, but this restricts incorporation of additional cytolytic and immunogenic transgenes in these viruses due to genetic cargo constraints. Moreover, OVs have so far not been engineered to target specific CAF subsets. Since CAFs exert diverse functions, both tumor-promoting and tumor-controlling, it is of considerable interest to study the natural tropism of wildtype or bioselected OVs towards CAFs. Natural GI CAF tropism has currently only been described for vesicular-stomatitis virus (VSV), but this virus is mostly studied in the pre-clinical setting [1, 13]. Therefore, we investigated the tropism of two commonly used OVs, adenovirus and reovirus, towards primary GI fibroblasts derived from human oesophageal, gastric, duodenal and pancreatic carcinomas. We show that GI fibroblasts are susceptible to type 3 Dearing (T3D) strain R124 and bioselected mutant reovirus (*jin*-3) infection but not adenovirus (Ad5-Δ24). Therefore, we focused on elucidating the requirements needed for efficient fibroblast infection by reoviruses. In short, we demonstrate the existence of fibroblasts expressing JAM-A, the reovirus entry receptor [14], in all GI cancer types investigated. JAM-A expression was predictive for efficient OV infection of fibroblasts and also appears to help in dissemination of the virus in tumor-fibroblast multicellular models. Finally, we show that JAM-A is not only required for productive reovirus infection but is also involved in apoptosis induction via signaling through the C-terminal PDZ-domain. Altogether, our data show the presence of a JAM-A expressing fibroblast subset in both human and murine GI cancers that is amenable to infection and apoptosis induction by oncolytic reovirus. This highlights an additional anti-tumor effect of oncolytic reovirus, next to direct lysis of tumor cells, and this natural CAF tropism could be potentially exploited to select GI cancer patients for OV therapy based on the JAM-A expression of both the tumor and stromal cells.

## Materials & methods

### Primary cell and cell line culture

Primary fibroblasts were derived from human GI cancers and adjacent tissue according to the Code of Conduct for Responsible Use of human tissues or after written informed consent was obtained. The regional Medical Ethics Assessment Committee (METC) approved use of this material (registration number: B21.073). Primary fibroblast isolation was performed by mincing tumor tissue or adjacent normal tissue, obtained from patients with the appropriate consent, into small fragments and subsequent digestion with a mix (3:1 ratio) of collagenase (Gibco/Thermo Fisher Scientific, Leiden, The Netherlands) and dispase II (Roche, Basel, Switzerland) for 2 h at 37 °C. Subsequently, cells were expanded and tested via qPCR for the presence of general fibroblast marker expression (collagen type 1 α1 (Col1α1) and alpha smooth muscle actin (αSMA)) and absence of markers of epithelial, endothelial and immune origin (E-Cadherin, CD31 & CD45, respectively). Primary fibroblasts and the hPS1 pancreatic stellate cell (PSC) line [15] (kindly provided by H. Kocher, University of London, London, England) were cultured in Dulbecco’s modified Eagle’s medium (DMEM)/F12 (Thermo Fisher Scientific) supplemented with 8% fetal calf serum (FCS), 100 IU/mL penicillin and 100 μg/mL streptomycin (all Thermo Fisher Scientific). Murine fibroblasts from pancreatic KPC tumors were isolated and cultured as described previously [16].

Culture of primary tumor cell lines, organoids and organoid-CAF co-cultures was performed as described previously [17]. In short, suspension co-cultures were obtained by mixing patient-derived organoids (PDOs) and primary fibroblasts at ratios of 500:4000 (PDO:CAF), respectively. Single cells after trypsinization were homogenously mixed in fibroblast medium containing 1% Matrigel, plated in ultra-low attachment 96-well round-bottom plates (Corning), centrifuged (1200 RPM, 1 min) and incubated overnight (37 °C, 5% CO2). The formed mini-tumors (MTs) were subsequently pooled and transferred to ultra-low attachment 6-wells plates (Corning) after which infection with oncolytic viruses was performed.

The HT29 colorectal cancer and 911 embryonic retinoblast-derived cell lines were cultured in high-glucose DMEM (Invitrogen), supplemented with 100 IU/mL penicillin and 100 μg/mL streptomycin and 8% FCS (Invitrogen, Breda, the Netherlands).

All cells were cultured at 37 °C and 5% CO_2_. Mycoplasma tests were performed regularly by PCR and were negative throughout the duration of the experiments.

### Lentiviral transductions and transgenic cell lines

Third-generation packaging vectors and HEK293T cells were used for the generation of lentiviral particles [18]. Lentiviral expression plasmids containing the full-length JAM-A open reading frame (ORF) (pLV-fullJAM-A) as well as a vector that lacks the cytoplasmic C-terminus (kindly provided by Diana van den Wollenberg, Dept. of Cell and Chemical Biology, LUMC) were described previously [19]. The PDZ-domain mutant was generated by *DpnI*-mediated site-directed mutagenesis [20] of the pLV-fullJAM-A vector by introducing a premature stop codon at position 295 (p295^S>*^), prior to the PDZ motif (-SFLV), using primers 5′-ggagaattcaaacagacctaatcattcctggtgtaatcc-3′ (forward) and 5′-ggattacaccaggaatgattaggtctgtttgaattctcc-3′ (reverse). Transduced cells were selected and cultured throughout the experiments with neomycin (400 µg/ml; Fisher Scientific). After transduction of hPS1 fibroblasts, membrane surface expression of the extracellular part of JAM-A was verified for all three constructs using flow cytometry.

For generation of JAM-A KO primary fibroblasts, sgRNA 5′- caccgTCGGGAGCCTGATCGCGATG-3′ (lowercase nucleotides are complementary to the *BsmBI-*restriction site) was cloned into *BsmBI*-digested pLentiCRISPRv2 [21] (Addgene: #98290), in which hSpCas9 and the guide RNA targeting JAM-A are co-expressed from the same vector. Lentiviral particles were generated and primary fibroblasts were subsequently transduced. After lentiviral transduction, genetic ablation of JAM-A was confirmed by flow cytometry. Cells were selected and cultured with 2 µg/ml of Puromycin (Sigma-Aldrich, Zwijndrecht, The Netherlands) for the duration of the experiments.

To fluorescently label KPC3 tumor cells and KPC-derived CAFs, they were lentivirally transduced with pLentiPGK Hygro DEST H2B-mCerulean3 (Addgene: #90234, KPC3 tumor cells) and pLentiPGK Hygro DEST H2B-mRuby2 (Addgene: #90236, CAFs). Cell selection and subsequent culture was performed using 400 µg/ml hygromycin (Gibco).

### Flow cytometry

For cell surface staining, cells were harvested and washed twice with FACS buffer, consisting of PBS/0.5% bovine serum albumin (BSA, Sigma) and 0.05% sodium azide (Pharmacy LUMC, Leiden, The Netherlands). Cells were incubated with rabbit anti-human JAM-A (EPR23244-12, Abcam) at 4 °C for 45 min. Subsequently, cells were washed twice with FACS buffer and incubated with goat anti-rabbit-PE (Jackson ImmunoResearch Europe Ltd, United Kingdom) at 4 °C for 45 min. For staining of β1-integrin, a directly PE-conjugated anti-human β1-integrin (MAR4; BD biosciences, CA, USA) was used. Samples were measured on a LSR-II (BD biosciences) and data were analyzed with FlowJo v10.6.1 software (BD biosciences).

### Western blot analysis

Total cell lysates were generated in RIPA buffer (50 mM Tris pH 7.5, 150 mM NaCl, 0.1% SDS, 0.5% DOC, 1% NP40), supplemented with complete mini protease inhibitor cocktail (Roche Applied Science, Penzberg, Germany). For the analysis of phosphorylated proteins, a commercial RIPA buffer (Pierce), supplemented with protease inhibitors (Roche), 50 U/mL benzonase (Santa Cruz) and sodium fluoride (Merck), was used to generate the lysates. Samples were cleared from cellular debris by centrifugation (13,000 RPM, 4 °C, 5 min). Protein concentrations were measured by a Pierce^TM^ BCA kit (Thermo Scientific, Rockford, IL, USA). Lysates were denatured by adding Laemmli sample buffer containing 20 mM DTT and heating for 5 min at 95 °C. Equal amounts of protein (10-30 µg) were separated by gel electrophoresis on 10% SDS-polyacrylamide gels, and transferred onto 0.2 μm nitrocellulose membranes using the Trans-Blot Turbo Transfer System (Bio-Rad). Membranes were blocked in TBS, supplemented with 0.1% Tween20 (TBST) and 10% milk or 10% BSA. Antibodies were diluted in TBST containing 5% milk, or in immuno booster (Takara) for the detection of phosphorylated proteins. Primary antibodies were incubated overnight at 4 °C, and secondary antibodies for 60 min at room temperature. Blots were washed with TBST.

The following antibodies were used: mouse anti-reovirus σ3 (4F2, Developmental Studies Hybridoma Bank, developed under the auspices of the NICHD and maintained by the University of Iowa, Department of Biology, Iowa City, IA, USA) [22], rabbit anti-phosphorylated MLKL (EPR9514, Abcam), rat anti-MLKL (MABC604, Merck Millipore), and mouse anti-vinculin (Sigma, V9131). Proteins were visualized using the Odyssey CLx Imaging System (LI-COR Biosciences). As a positive control for necroptosis induction, a cocktail was used that consists of TNF-alpha (InvivoGen Europe), BV6 (AbMole), and Z-VAD-FMK (Bachem AG).

### RNA isolation and RT-qPCR analysis

Total RNA was isolated using the NucleoSpin RNA isolation kit (Macherey-Nagel, Düren, Germany) according to manufacturer’s instructions. cDNA was synthesized with the RevertAid First strand cDNA synthesis kit (Thermo Fisher Scientific) using 0.5–1.0 µg as RNA input. RT-qPCR was performed with SYBR Green Master mix (Bio-Rad laboratories, Nazareth, Belgium) using the iCycler Thermal Cycler and iQ5 Multicolour Real-Time PCR Detection System (Bio-Rad). Target genes were amplified using specific primers (supplementary Table 1). The ΔCt or ΔΔCt method was applied to calculate the levels of gene expression, relative to the reference gene (β-actin) or a control condition, respectively. Reovirus S4 10log copy numbers were determined using a standard curve, consisting of serial dilutions of plasmid pcDNA_S4. Copy numbers were calculated according to a described formula for rotavirus NSP3 quantification [23].

### Oncolytic viruses

The wild-type type 3 Dearing (T3D) reovirus strain R124 was isolated by plaque purification from a heterogenous T3D stock obtained from ATCC (VR-824), and propagated as described previously [24]. The *jin*-3 mutant reovirus was isolated from U118MG cells upon infection with wild-type T3D. The genomes of the R124 and *jin*-3 viruses have been fully sequenced. GenBank IDs of the R124 segments are: L1 GU991659; L2 GU991660; L3 GU991661; M1 GU991662; M2 GU991663; M3 GU991664; S1 GU991665; S2 GU991666; S3 GU991667; S4 GU991668. Ad5-Δ24 is based on human serotype 5 (Ad5) and constructed using AdEasy technology. It contains a 24-nucleotide deletion (923-946) in E1A, encoding the amino acid sequence that is implicated to be vital for binding of the tumor-suppressive Retinoblastoma protein (pRb) [25]. As a result, the virus exerts tumor-selective replication [26].

Infectious virus titers were determined by plaque assays on 911 cells, as previously described [24]. All experiments were performed using CsCl-purified virus stocks. For purification, a freeze-thaw lysate containing virus particles was incubated with 0.1% Triton (Sigma-Aldrich, Zwijndrecht, The Netherlands) and 25 units/ml Benzonase (Santa Cruz, Bio-Connect B.V. Huissen, The Netherlands) for 15 min on ice followed by 15 min at 37 °C. After two extractions with Halotec CL10 (FenS B.V. Goes, The Netherlands), the cleared lysate was loaded onto a discontinuous CsCl gradient (1.45 and 1.2 g/cm^3^ in phosphate-buffered saline (PBS)). After centrifugation in a SW41 rotor (Beckman Coulter, Woerden, The Netherlands) at 20,000 *g* for 14 h at 4 °C, the lower band containing the infectious particles was harvested and desalted in an Amicon Ultra 100 K device according to the manufacturer’s protocol (Millipore, Merck Chemicals BV, Amsterdam, the Netherlands). The CsCl-purified reoviruses were recovered in reovirus storage buffer (RSB: 10 mM Tris-HCl, pH 7.5, 150 mM NaCl, 10 mM MgCl_2_ • 6 H_2_O) and stored at 4 °C until use. The CsCl-purified adenoviruses were recovered in adenovirus storage buffer (ASB: 140 mM NaCl, 5 mM Na_2_HPO_4_.2H_2_O, 1.5 mM KH_2_PO_4_, 5% sucrose, pH 7.8) and stored at −80 °C until use. An aliquot was used for OD_260_ measurement prior to storage to calculate the amount of viral particles.

### Cell viability assays

To examine the viability of cells upon oncolytic virus infection, WST-1 reagent (Roche, Woerden, The Netherlands) was employed. In 96-wells plates, cells were seeded at 5–20 × 10^3^ cells/well (5 wells per condition) in their respective culture media. Virus infections were performed in medium containing 2% FCS. Upon exposure to reoviruses R124 or *jin*-3, or Ad5-Δ24 at varying MOIs (0,01–10) for 3–5 days, the WST-1 read-out was performed. In short, 5 µl of WST-1 reagent per well was supplemented with 100 µl of fresh medium and added to each well. After incubation for 1–2 h, the OD450 values were measured and the percentage of cell viability was calculated by dividing the OD450 values of the virus-treated wells by the values of the mock condition. Negative values were manually adjusted to 1% to allow for plotting on linear as well as logarithmic scales. Crystal violet staining was performed according to the manufacturer’s protocol (Sigma). Briefly, 1 × 10^5^ cells were seeded in 12-well plates and infected with different MOIs (0.1, 1 and 10) of reovirus wildtype R124 and mutant *jin*-3 for 2 days. Subsequently, reovirus-infected cells were fixed in with 4% PFA (Added Pharma, Oss, The Netherlands), washed twice with PBS and stained for 15 min with crystal violet. After subsequent washing with PBS, whole wells were imaged using the Cytation Microplate Reader (Biotek, Winooski, VT, USA).

### Caspase (apoptosis) assays

In 96-wells plates, hPS1 cells were seeded at 1 × 10^4^ cells/well in triplicate. Reovirus R124 or *jin*-3 infections were performed at a MOI of 10. To measure caspase 3/7 activity, a Caspase-Glo 3/7 assay (Promega, Leiden, The Netherlands) or CellEvent Caspase 3/7 Green Detection assay (Invitrogen, Breda, The Netherlands) was performed according to the manufacturer’s protocol. The Caspase-Glo 3/7 read-outs were performed at 48 h post-infection on a PerkinElmer’s VictorX3 (PerkinElmer, Groningen, The Netherlands) multilabel plate reader. The fold changes over the mock were calculated by dividing the values of the virus-treated wells over the values of the mock-exposed wells. The CellEvent Caspase 3/7 Green Detection assay was measured on a Cytation Microplate Reader (Biotek) at 500 nm excitation and 530 nm emission at intervals of 30 min throughout the course of the experiment.

### Immunohistochemical and immunofluorescent staining

Four-micrometers sections were deparaffinized and processed for immunohistochemistry (IHC) or immunofluorescent (IF) staining. For IHC, sections were blocked in 0.3% hydrogen peroxidase (H_2_O_2_, Merck, Darmstadt, Germany) in methanol for 20 min. Next, IHC and IF slides were rehydrated, and antigen retrieval was performed by boiling in 0.01 M sodium citrate (pH 6.0) for 10 min. Slides were washed and incubated with mouse anti-sigma 3 (4F2, Developmental Studies Hybridoma Bank, Iowa, IA, USA), mouse-anti JAM-A (2E3-1C8, Abnova, Taipei, Taiwan), mouse anti-pan-cytokeratin (PKC-26, Sigma) and/or rabbit anti-human vimentin (D21H3, Cell signaling, Leiden, The Netherlands) antibodies diluted in 1% BSA/PBS overnight at room temperature in a humidified box. The next day, slides were incubated with appropriate biotinylated secondary antibodies (Agilent technologies, CA, USA) or anti-mouse-Alexa 488 (Thermo Fisher Scientific) and anti-Rabbit-alexa 568 (Thermo Fisher Scientific). For immunofluorescent staining, slides were mounted with ProLong™ Gold Antifade Mountant (Thermo Fisher Scientific) including DAPI. For IHC, slides were incubated with Vectastain complex (Vector Laboratories, CA, USA) at room temperature for 30 min. Staining was visualized with the Dako Liquid DAB+ Substrate Chromogen kit (Agilent Technologies) for 10 min. Nuclei were counterstained with Mayer’s Hematoxylin (Merck) and slides were rinsed in tap water, dehydrated, and mounted using Entellan (Merck). Images for IHC were obtained with an Olympus BX51 Light Microscope equipped with an Olympus DP25 camera. For IF, images were taken with a Leica DMi8 or Leica DM6B microscope (Leica). All images were analyzed using Fiji software.

### Animal experiments

All performed animal procedures were approved by the Central Authority for Scientific Procedures on Animals. For all experiments, 8–12 weeks old C57BL/6JIco mice of both genders were used. Sample sizes were determined based on variation observed in previous experiments. Mice were distributed over groups with equal tumor volumes and were not randomized. Treatment was not blinded. Subcutaneous injection of KPC3-luc2 (1,0 x 10^5^/mouse) in the flank was performed at day 0 after which tumor size was followed up by caliper measurements. When tumor size reached between 50–200 mm^3^ (approximately day 13), 10^9^ PFU reovirus or solvent control (reovirus storage buffer) in control animals was injected intratumorally. Mice were sacrificed at a predefined tumor volume of 1500 mm^3^ or when weight loss exceeded 20% of baseline body weight. Subsequently, subcutaneous tumors were explanted and processed for histology.

### Statistical analysis

Data are presented as means ± standard deviation from representative experiments of independent replicates. Unpaired Student *t*-tests were used to compare 2 groups. Differences between more than 2 groups were measured using 1-way analysis of variance (ANOVA) or 2-way ANOVA, depending on the amount of variables, and corrected for multiple testing. The correlation between JAM-A expression and cell viability was investigated using the Pearson correlation coefficient. All analyses were performed using GraphPad Prism software (San Diego, CA, USA). P values of 0.05 or less were considered statistically significant.

## Results

### Reoviruses R124 and jin-3, but not adenovirus Ad5-Δ24, induce cell death of primary GI cancer-derived fibroblasts

**Fig. 1:**
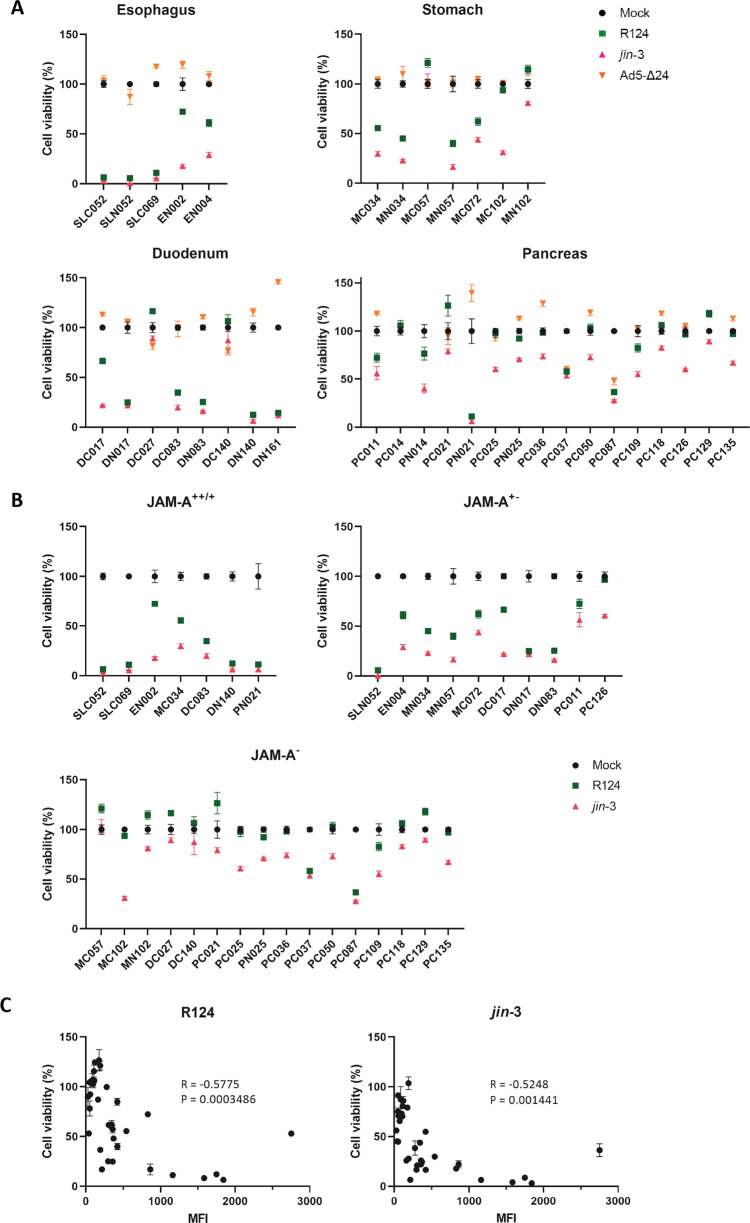
Targeting of GI fibroblasts with oncolytic viruses. **A** Oesophageal, gastric, duodenal and pancreas fibroblasts were infected with the indicated OVs at MOI 10 after which cell viability was determined 5 days post-infection. **B** GI fibroblast cell viability is shown according to the expression of JAM-A as determined by flow cytometry (++: MFI > 1500, +: MFI 500–1500, +/−: MFI 250-500, −: MFI < 250). **C** Pearson’s correlation of JAM-A MFI and cell viability as assessed by flow cytometry and WST-1 assay. Left panel shows correlation for R124 (R = −0.5775, *p* = 0.0003) and right panel for *jin-3* (R = −0.5248, *p* = 0.0014). MFI = mean fluorescence intensity, C = CAF, N = tumor-adjacent.

To investigate the ability of OVs to induce cell death in CAFs, patient-derived fibroblasts (supplementary figure 1A, B) from different primary GI cancers and adjacent tissue (oesophagus, gastric, duodenum, pancreas) were infected with reovirus (R124 and *jin*-3) and adenovirus (Ad5-Δ24). Five days post-infection, considerable cell death was observed in the majority of the fibroblast cultures infected with either R124 or *jin*-3 reoviruses (Fig. 1A). Ad5-Δ24 however did not induce cell death in the vast majority of fibroblasts tested, despite the presence of the cognate entry receptor Coxsackie and Adenovirus Receptor (CAR) (supplementary table 2). In contrast, a control tumor cell line (BxPC3) was killed by Ad5-Δ24 (supplementary figure 2), indicating that this virus has oncolytic capacities but is not capable of inducing cell death in stromal cells. Given the limited sensitivity of GI fibroblasts to adenovirus-induced cell death, this virus was therefore not further analysed.

Intriguingly, fibroblasts isolated from different sites of the GI tract did show differential sensitivity to reovirus-induced cell death. Oesophageal, gastric and duodenum-derived fibroblasts were most sensitive to both R124 and *jin*-3, while pancreatic fibroblasts were predominantly refractory to R124 and, to a lesser extent, *jin*-3-induced cell death. The observed differences in reovirus-induced CAF killing imply that there is variation in virus susceptibility between fibroblasts derived from different organs. Since a key determinant in productive infection is viral entry, we determined the expression of the reovirus entry receptor JAM-A [14] on these fibroblasts and grouped them accordingly (supplementary table 2). Both fibroblasts isolated directly from the tumor as well as adjacent tissue were found to express JAM-A. There was a significant correlation (Fig. 1B, C, Pearson’s R = −0.58 and R = −0.52 for R124 and *jin*-3, respectively) between the surface expression of JAM-A and susceptibility to reovirus-induced cell death. Of note, *jin-3* was still partially effective in inducing cell death in JAM-A negative fibroblasts, compatible with the capacity of this mutant virus to also enter cells in the absence of JAM-A [24]. Collectively, these data show that oncolytic reovirus has a natural capacity of inducing cell death in a subset of GI fibroblasts and that this correlates with the expression of JAM-A.

### Reovirus infection and killing of fibroblasts is mediated by JAM-A expression

**Fig. 2:**
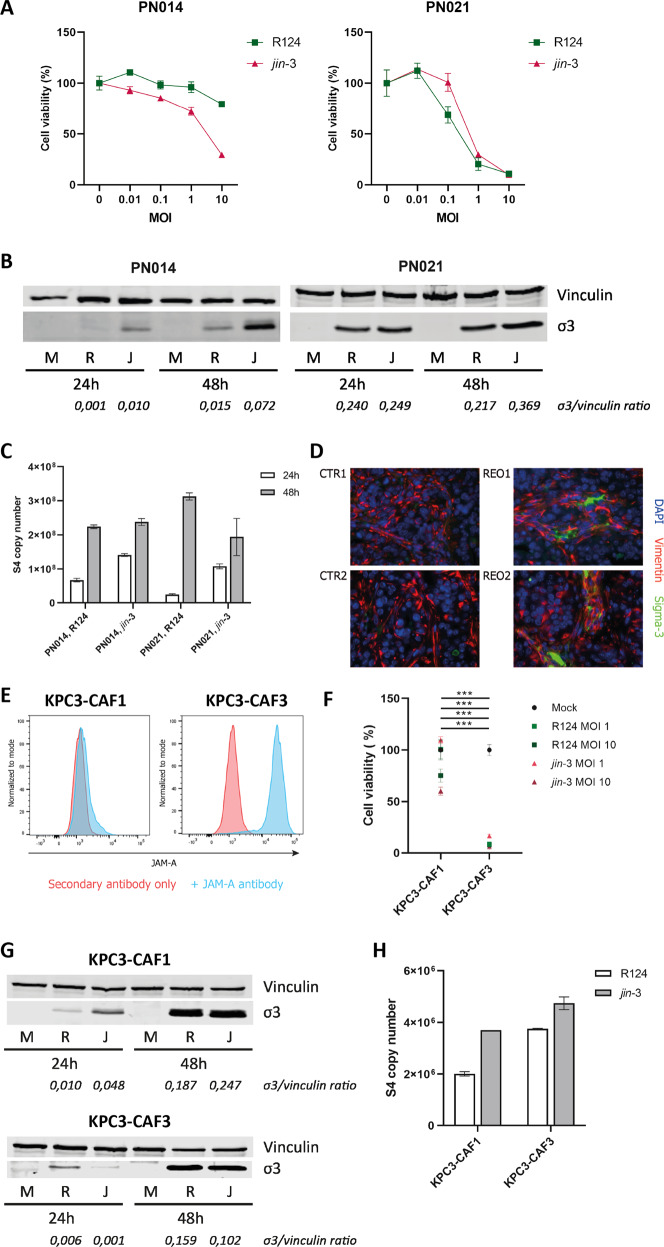
Infection and cell death of fibroblasts according to JAM-A status. **A** Cell viability of JAM-A negative (PN014) and JAM-A positive (PN021) human pancreatic fibroblasts infected with R124 and *jin-3* at different MOIs. **B** Western blot for σ3 protein in JAM-A negative and JAM-A positive human fibroblasts infected with R124 and *jin-3* for indicated time points. M = mock, R = R124, J = *jin-3* **C** S4 copy number as determined by RT-qPCR in JAM-A negative and JAM-A positive human fibroblasts infected with R124 and *jin-3* for indicated time points. **D** Immunofluorescent staining of KPC3 subcutaneous tumors for vimentin and σ3 protein after reovirus infection (REO1, REO2) or mock control (CTR1, CTR2). **E** Flow cytometry of murine CAF cultures derived from KPC3 tumors for expression of JAM-A. **F** Viability of JAM-A negative (KPC3-CAF1) and JAM-A positive (KPC3-CAF3) murine fibroblast cultures 5 days post-infection with R124 or *jin-3*. Two-way ANOVA for all different virus conditions, *** = *P* ≤ 0.0001. **G** Western blot for σ3 protein in JAM-A negative and JAM-A positive murine fibroblasts infected with R124 and *jin-3* for indicated time points. M = mock, R = R124, J = *jin-3* **H** S4 copy number as determined by RT-qPCR in JAM-A negative and JAM-A positive murine fibroblasts infected with R124 and *jin-3* for indicated time points.

To further study the reovirus infectious cycle in fibroblasts, we studied fibroblasts from two PDAC patients, that differed in their JAM-A expression (supplementary figure 3). PDAC was chosen for this study due to it being a prototypical example of a desmoplastic GI tumor, with stromal abundance and poor patient survival. As expected, killing of the fibroblasts was strongly dependent on the expression of JAM-A (Fig. 2A). Subsequently, we looked at the expression of viral RNA and protein in these JAM-A positive and negative primary fibroblasts as an indicator of viral replication. Productive infection, as indicated by detection of the viral outer capsid protein sigma-3 (σ3), was observed in the JAM-A positive and to a lower extent negative fibroblasts (Fig. 2B). A similar trend was observed for the expression of the viral S4 segment, used as a measure of viral genome replication. S4 expression was detected in both JAM-A positive and negative fibroblasts, but was clearly higher in JAM-A expressing fibroblasts 48 h after infection (Fig. 2C). To study the extent to which our findings can be recapitulated in an in vivo model, the presence of JAM-A expressing fibroblast subsets was investigated in the KPC model for PDAC. Subcutaneously implanted tumors were injected with 10^9^ PFU reovirus when palpable and when reaching predefined endpoints, mice were sacrificed and explanted tumors were analysed. Immunofluorescent staining of tumor tissue revealed the presence of σ3 protein deposits in areas where fibroblasts (defined as spindle-shaped, vimentin-positive cells) were located (Fig. 2D). To further study if KPC-derived CAFs would be susceptible to reovirus-induced cell death, primary fibroblasts were isolated from (uninfected) KPC tumors. KPC-CAFs were characterized by high expression of Col1α1and αSMA (supplementary figure 4A). Moreover, these CAFs expressed wildtype KRAS (supplementary figure 4B) confirming they were not of epithelial origin (tumor cells contain an engineered mutant KRAS gene). Isolation of CAFs from different parts of the tumors resulted in CAF cultures with varying expression of JAM-A (Fig. 2E). In these murine CAFs, JAM-A expression was also associated with sensitivity to reovirus-induced cell death (Fig. 2F). However, similar to the findings in human CAFs, σ3 protein, and gene expression was detected in both murine CAFs (Fig. 2G–H**)**, indicating infection can occur also in JAM-A negative fibroblasts. Taken together these data indicate that murine CAFs are sensitive to reovirus infection in vitro and in vivo and show JAM-A dependent cell death.

**Fig. 3:**
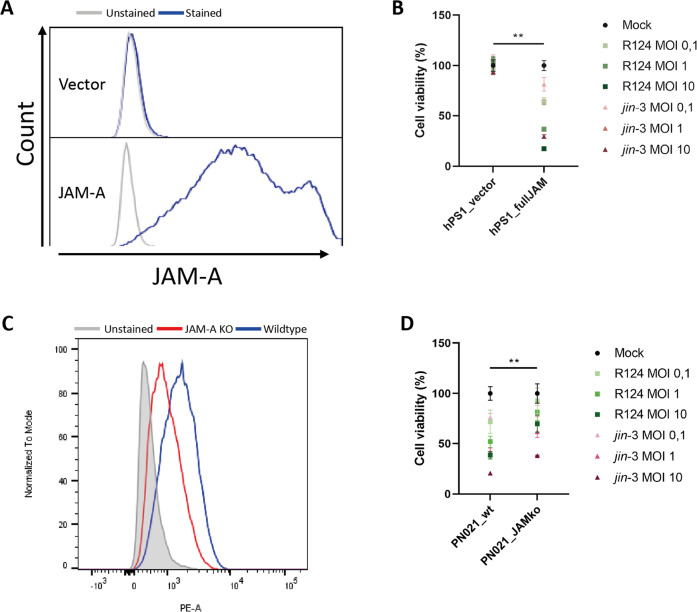
Overexpression and ablation of JAM-A in pancreatic fibroblasts. **A** JAM-A expression on hPS1 pancreatic stellate cells transduced with full-length JAM-A cDNA or vector control. **B** Cell viability of hPS1 vector control (hPS1_vector) or hPS1 expressing the full-length JAM-A cDNA (hPS1_fullJAM) after infection with R124 or *jin-3* at indicated MOIs, 3 days post-infection. Student’s *T*-test, ** = *P* ≤ 0.01. **C** JAM-A expression on primary pancreatic fibroblasts (Wildtype) or CRISPR/Cas9-mediated JAM-A KO fibroblasts. **D** Cell viability of wildtype or JAM-A KO fibroblasts after infection with R124 or *jin-3* at indicated MOIs, 5 days post-infection. Student’s *T*-test, ** = *P* ≤ 0.01.

To further validate the involvement of JAM-A in reovirus-mediated CAF killing, we ablated and overexpressed JAM-A in pancreatic fibroblasts. Overexpression of JAM-A in the JAM-A negative PSC line hPS1 [15] (Fig. 3A), naturally resistant to reovirus-induced cell death, led to strongly increased cell death induced by reovirus infection and already resulted in productive infection, indicated by σ3 expression, at low MOI (Fig. 3B, supplementary figure 5). Conversely, genetic ablation via CRISPR/Cas9-mediated knock-out of JAM-A (Fig. 3C) in primary pancreatic CAFs led to a significant decrease in cell death (Fig. 3D) upon reovirus infection. Altogether, these data indicate that JAM-A facilitates, but is not strictly necessary for reovirus entry, and additionally mediates reovirus-induced cell death in GI fibroblasts.

### JAM-A expression on fibroblasts facilitates reovirus infectivity of tumor-fibroblast co-cultures

**Fig. 4:**
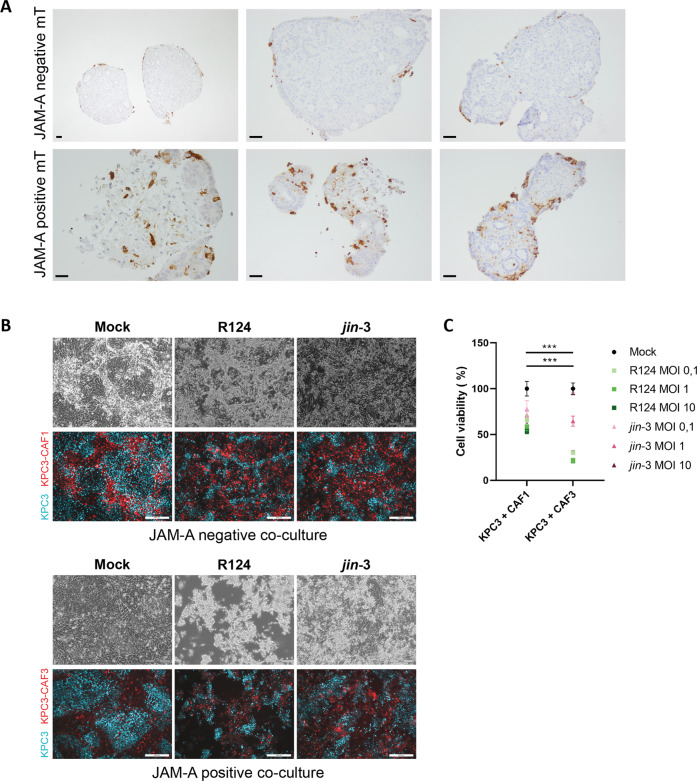
JAM-A expressing fibroblast-tumor co-cultures show enhanced reovirus infectivity. **A** Human organoid-fibroblast co-cultures consisting of JAM-A negative or JAM-A positive fibroblasts were infected with R124 at MOI 10. 7 days post-infection, organoid-fibroblast co-cultures were embedded and stained for σ3 protein. Three different JAM-A negative (top panel) and JAM-A positive (bottom panel) are shown. Scale bar, 50 μm. **B** 2D co-cultures of KPC3 tumor cells (mCerulean) with JAM-A negative (CAF1) or JAM-A positive (CAF3) fibroblasts (mRuby) after infection with R124, *jin-3*, or mock control two days post-infection. Scale bar, 500 μm. **C** Cell viability of KPC3 tumor-fibroblast co-cultures two days post-infection. Two-way ANOVA for R124 and *jin-3* MOI 10 conditions, *** = *P* ≤ 0.0001.

Having established that PDAC fibroblasts are amenable to reovirus infection, we subsequently investigated the effect of the JAM-A status of fibroblasts on reovirus infectivity in tumor-fibroblast co-cultures (supplementary figure 6). Human PDAC 3D-mini tumor (mT) models were generated that consisted of the same pancreatic cancer organoids combined with either JAM-A positive or negative primary, patient-derived CAFs. After exposure to reovirus for 5 days, these organoid-fibroblast co-cultures were embedded and stained for the presence of the viral σ3 protein. Co-cultures generated with JAM-A positive fibroblasts showed increased σ3 staining compared to their JAM-A negative counterparts, indicating a potential role for JAM-A expressing fibroblasts in promoting virus spread throughout the tumor (Fig. 4A). In line with this, murine co-cultures consisting of the KPC3 tumor cell line and JAM-A positive CAFs showed increased signs of killing compared to co-cultures containing JAM-A negative fibroblasts (Fig. 4B). Moreover, overall cell viability was decreased in co-cultures holding JAM-A expressing fibroblasts as compared to JAM-A negative fibroblasts (Fig. 4C). Combined with the data on the role of JAM-A in reovirus-mediated CAF killing, this points to a potential bimodal mechanism of action of targeting JAM-A expressing fibroblasts with reovirus that would enable (1) lysis of JAM-A expressing desmoplastic stromal cells and (2) serve as a conduit for OV replication and subsequent infection of desmoplastic-adjacent tumor cells.

### JAM-A promotes reovirus-induced apoptosis in fibroblasts through the C-terminal PDZ domain

**Fig. 5:**
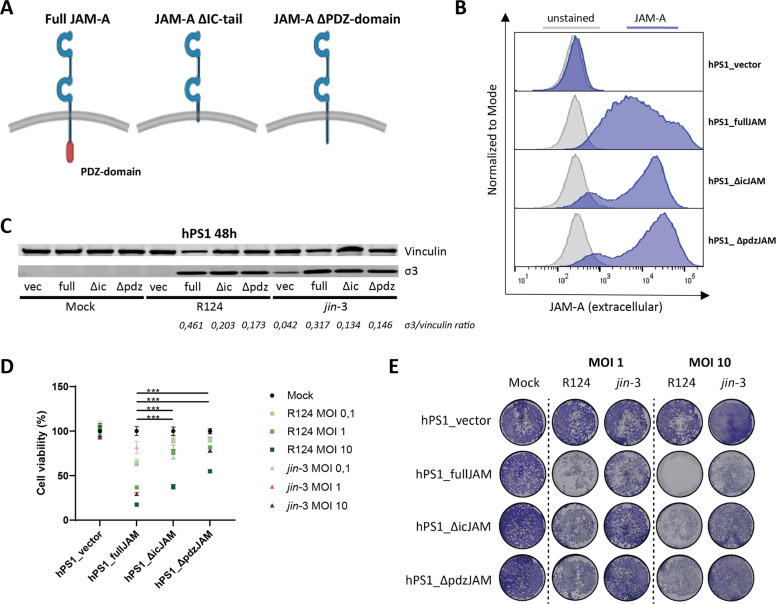
Fibroblasts expressing JAM-A cytoplasmic tail mutants show decreased sensitivity to reovirus-induced cell death. **A** Overview of the different JAM-A constructs generated and expressed in hPS1 pancreatic stellate cells. **B** Cell surface expression of JAM-A on the hPS1 cells expressing vector control, full-length JAM-A and JAM-A cytoplasmic tail mutants. **C** Western blot for σ3 protein in the different JAM-A constructs infected with R124 and *jin-3* for 48 h. **D** Cell viability of the hPS1 cells expressing the different JAM-A constructs after 72 h of infection with R124 or *jin-3* at indicated MOIs. Two-way ANOVA for R124 and *jin-3* MOI 10 conditions, *** = *P* ≤ 0.0001 **E** Crystal violet staining of the hPS1 cells expressing the different JAM-A constructs after 72 h of infection with R124 or *jin-3* at indicated MOIs.

The enhanced cytolytic capacity of reovirus in JAM-A expressing fibroblasts could be a result of more efficient entry of reovirus in JAM-A expressing fibroblasts and thereby cell death or, alternatively, direct lysis through JAM-A dependent signaling following reovirus binding. To establish whether JAM-A signaling is directly involved in reovirus-induced cell death of fibroblasts, we generated JAM-A mutants that either completely lack the cytoplasmic tail (ΔicJAM) or only the terminal part of the cytoplasmic tail that harbors the PDZ-domain (ΔpdzJAM). The PDZ domain is involved in interacting with all currently known JAM-A binding partners [27] (Fig. 5A). Lentiviral transduction of hPS1 fibroblasts with the full-length or one of the mutant JAM-A cDNAs led to similar cell surface expression levels of the extracellular domain of JAM-A, which is required for reovirus binding [14, 28] (Fig. 5B). 48 h post-infection with either R124 or *jin*-3, the amount of intracellular virus was determined by detecting the amount of the viral σ3 protein. No significant differences in σ3 expression were observed between cells expressing the different JAM-A constructs, indicating that reovirus entry is most likely not disturbed by perturbing the cytoplasmic signaling domain of JAM-A (Fig. 5C). To further validate this, we analyzed the expression of surface β1-integrin, since it has been previously shown that this is required for internalization of reovirus in cells expressing a mutant JAM-A that lacks the intracytoplasmic tail [29]. Flow cytometric analysis revealed that hPS1 fibroblasts expressing the different JAM-A constructs all expressed β1-integrin (supplementary figure 7), indicating that the prerequisites for reovirus internalization are present for all JAM-A constructs. In contrast, WST-1 cell viability assays at the same time point showed a strong decrease in reovirus-induced cell death for both JAM-A mutants (ΔicJAM and ΔpdzJAM). This was most apparent at lower MOI (0.01–1) (Fig. 5D). These findings were confirmed by crystal violet staining of viable fibroblasts after reovirus infection (Fig. 5E), where a clear decrease in reovirus-induced cell death was observed in both JAM-A mutant fibroblasts. These data indicate that the intracellular domain of JAM-A is important in inducing reovirus-induced cell death.

**Fig. 6:**
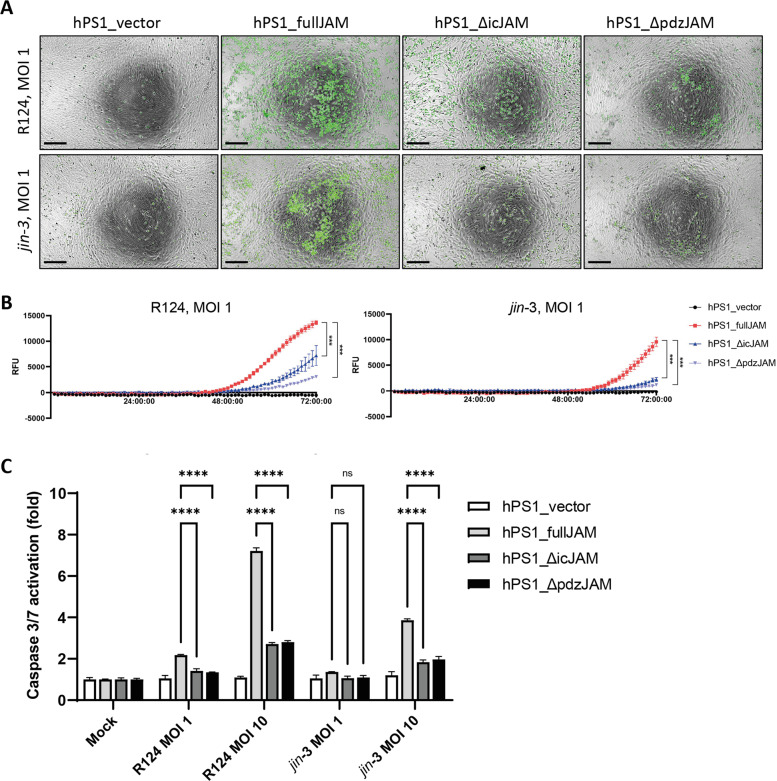
The C-terminal PDZ-domain of JAM-A mediates reovirus-induced apoptosis in fibroblasts. **A** Real-time microscopy (T = 72 h) of Caspase3/7 cleavage in hPS1 pancreatic stellate cells expressing different JAM-A constructs after infection with R124 (top row) or *jin-3* (bottom row).. Scale bar, 250 μm. **B** Quantification of Caspase3/7 signal in Relative Fluorescence Units for the entire duration of the experiment (72 h). Two-way ANOVA, *** = *P* ≤ 0.0001 **C** Relative caspase 3/7 activation in hPS1 pancreatic stellate cells expressing different JAM-A constructs as compared to the vector control.

Since virus-induced cell death can occur via multiple routes [30], we sought to establish the mechanism of reovirus-mediated fibroblast killing. To this end, cleavage of caspase 3/7, a hallmark of apoptosis, was visualized using live-cell immunofluorescence microscopy. Fibroblasts with stable expression of the JAM-A constructs were exposed to a caspase-dependent reporter during the course of reovirus infection. Interestingly, hPS1 cells transduced with the JAM-A mutants displayed significantly less cleaved caspase 3/7 than their full length counterpart but more than the vector control (Fig. 6A, B; supplementary video 1). A Caspase-Glo 3/7 assay, measuring the processing of a luminogenic caspase 3/7 substrate, showed a similar trend as the real-time microscopy experiments (Fig. 6C). Necroptosis, another form of cell death described to be induced by reovirus infection [31], was also studied by investigating the phosphorylation of the pseudokinase mixed lineage kinase domain-like (MLKL) [32, 33]. No induction of MLKL phosphorylation was observed in hPS1 fibroblast upon reovirus infection or stimulation with the necroptosis-inducing mixture TBZ (supplementary figure 8). In conclusion, these data indicate that reovirus-induced fibroblast killing is partially dependent on direct induction of apoptosis via JAM-A signaling through the C-terminal PDZ domain and indicates that JAM-A is not merely an entry receptor, but directly involved in inducing cell death.

## Discussion

The mechanism of action of oncolytic reovirus is currently mainly thought to be reliant on the infection of tumor cells, which in the majority of GI cancers are vastly outnumbered by the amount of stromal cells. In this study, we show the ability of wildtype (R124) and bioselected (*jin*-3) reoviruses to also infect fibroblasts, the predominant stromal cell type in GI cancers. Expression of the reovirus entry receptor, JAM-A, was not a prerequisite but facilitated infectivity by reoviruses in both murine and human GI-cancer derived fibroblasts. Efficient reovirus-induced cell death was mainly observed in JAM-A+ fibroblasts and was partially dependent on signaling through the PDZ-domain. Finally, co-cultures of tumor cells and fibroblasts showed increased infectivity if the fibroblasts expressed JAM-A, suggesting a potential role of JAM-A expressing stromal cells for intratumoral viral transmission.

We investigated the natural tropism for fibroblasts of two members of different (oncolytic) virus families, *Reoviridae* (R124, *jin*-3) and *Adenoviridae* (Ad5-Δ24), that are currently studied in multiple clinical trials [1]. Intriguingly, multiple fibroblasts expressed the entry receptor for adenovirus, CAR, but were not susceptible to adenovirus-induced cell death. In contrast, almost all JAM-A expressing fibroblasts showed susceptibility to reovirus-induced apoptosis by both reoviruses. This discrepancy might be explained by the genetic constitution of Ad5-Δ24, since it contains a E1A^(Δ923-946)^ deletion [25] that is vital for suppression of Rb-mediated cell cycle progression. This makes this particular virus tumor-selective but might make it less suitable for targeting of stromal cells in which the Rb-pathway is still intact. In contrast, reovirus tumor-selectivity largely stems from activation of the epidermal growth factor receptor (EGFR)/Ras pathway in host cells [34], which has also shown to be activated in tumor-derived fibroblasts [35]. GI-cancer derived fibroblasts that express the viral entry receptor, JAM-A, thus appear to serve as a suitable host to fully complete the lytic reovirus infectious cycle. Importantly, fibroblasts derived directly from tumor or tumor-adjacent tissue both showed expression of JAM-A. In contrast, fibroblasts derived from a true non-cancerous origin, such as VH10 (skin) and hPS1 (pancreas) cell lines do not express JAM-A [24]. Of note, isolation of fibroblasts from normal pancreata destined for transplantation was unsuccessful. This raises the question whether tumor-adjacent fibroblasts should be considered normal, since previous research has also shown extensive (sub)mucosal alterations in tissue adjacent to the tumor site [36]. Of note, *jin*-3 has previously been shown to enter cells independent of JAM-A due to mutations in the S1 segment that enable viral entry through sialic acid [24]. In this study we also observed more viral replication and cell death in JAM-A negative fibroblasts infected by *jin*-3 as compared to wildtype reovirus R124. However, expression of JAM-A was still a strong predictor for reovirus-induced cell death by *jin*-3, indicating that this virus still utilizes this receptor when available. JAM-A receptor expression has previously been described on epithelial (tumor) cells [37–39], endothelial cells [39, 40], leukocytes [41, 42] but, to the best of our knowledge, not on human (cancer-associated) fibroblasts. In contrast, another member of the family of junctional adhesion molecules, JAM-C, has been reported to be expressed by primary human fibroblasts, but in this study JAM-A was not detected on human dermal, lung and corneal fibroblasts [43]. A recent study shows that CAFs in a murine model of melanoma (B16) can also express JAM-A [44] and are susceptible to reovirus-induced cell death. Our data show that in patients with oesophageal, gastric, duodenal and pancreatic cancer, a subset of primary fibroblasts express JAM-A. Thus, it would be of interest to further investigate primary fibroblasts isolated from different anatomical sites to see whether these also express JAM-A and are therefore likely to be amenable to reovirus-induced cell death. Moreover, identification of negative and positive regulators of JAM-A expression in fibroblasts would be of value in order to attempt to convert reovirus-resistant fibroblasts (or other cells in the TME) to a permissive state.

Interestingly, reovirus-induced cell death of GI fibroblasts was also mediated by the presence of JAM-A, which stimulated apoptosis. A previous study [14] showed a functional role for JAM-A in reovirus-mediated apoptosis induction but this was contradicted in a later report, where JAM-A mediated apoptosis was not observed [45]. Our results are in accordance with a functional role for JAM-A signaling in reovirus-mediated apoptosis induction and we show that this is mediated specifically via the C-terminal PDZ-domain of JAM-A. Importantly, we show that virus entry is not dependent on the PDZ-domain of JAM-A, as shown previously [29], and that the differences in apoptosis induction that are observed are thus not a consequence of impaired reovirus internalization. Discrepancies between our findings and the study by Danthi et al. [45], although using similar approaches, might stem from the host cell in which JAM-A cytoplasmic tail mutants were expressed, being either human fibroblasts or Chinese Hamster Ovary (CHO) cells [45], respectively. Possibly, a downstream JAM-A binding protein is involved in reovirus-induced apoptosis that is non-homologous between these species, providing an avenue for rationally dissecting the molecular components involved in this process.

Finally, having discerned the molecular requirements for reovirus-mediated fibroblast targeting, we investigated the effect of reovirus infection of tumor-fibroblast co-cultures and in vivo. Interestingly, we observed the presence of reovirus in fibroblasts in vivo and were also able to isolate both JAM-A negative and positive fibroblasts from murine KPC tumors. Human and murine tumor-fibroblast co-cultures revealed that the presence of JAM-A expressing fibroblasts correlates with increased reovirus dissemination, pointing to a potential role of JAM-A+ fibroblasts in serving as an intratumoral viral conduit. OV-mediated targeting of the tumor stroma therefore seems an attractive approach, to (1) disrupt the desmoplastic stromal barrier, enabling immune cell invasion and increased influx of therapeutics and (2) serve as a conduit for OV replication and subsequent infection of desmoplastic-adjacent tumor cells. The former mechanism has also been observed in a PDAC model in Syrian hamsters in which treatment with a bioselected adenovirus resulted in less-dense stroma, enhancing chemotherapeutic penetration of the tumor bed [46]. Interestingly, the latter mechanism has also been observed in patient-derived PDAC xenografts in which VSV-infected CAFs sensitized tumor cells to VSV infection by downregulation of the antiviral retinoic acid-inducible gene I (RIG-I) via production of fibroblast growth factor 2 (FGF2) [13].

In conclusion, our findings show the presence of JAM-A+ fibroblasts in a variety of human GI cancers and their predisposition to be targeted by oncolytic reoviruses. We highlight the role of JAM-A as being the viral entry receptor as well as a direct mediator of reovirus-induced apoptosis in human GI fibroblasts. Moreover, our results indicate that the presence of JAM-A+ fibroblasts results in increased infectivity of fibroblasts as well as adjacent tumor cells. Altogether, we show an additional anti-tumor effect of reovirus, next to direct lysis of tumor cells, and argue that this natural CAF tropism can be potentially exploited to select GI cancer patients for OV therapy based on the JAM-A expression status of both tumor cells and tumor-adjacent stroma.

## Supplementary information

Supplementary tables and figure legends

Supplementary figure 1

Supplementary figure 2

Supplementary figure 3

Supplementary figure 4

Supplementary figure 5

Supplementary figure 6

Supplementary figure 7

Supplementary figure 8

Supplementary video 1

## Data Availability

The data that support the findings of this study are available from the corresponding author upon reasonable request.
