# Supplementary material for: Gastrointestinal cancer-associated fibroblasts expressing Junctional Adhesion Molecule-A are amenable to infection by oncolytic reovirus
Source: Cancer Gene Ther. 2022 Jul 22;29(12):1918–29. doi: 10.1038/s41417-022-00507-9 (PMC9750869; doi:10.1038/s41417-022-00507-9)
Supplement: Supplementary file 1 — Supplementary tables and figure legends [file 41417_2022_507_MOESM1_ESM.docx]

*Supplementary tables*

| **Gene (h, human; m, murine)** | **Forward primer (5’ --- 3’)** | **Reverse primer (5’ --- 3’)** |
| --- | --- | --- |
| hβ-actin | GTTGTCGACGACGAGCG | GCACAGAGCCTCGCCTT |
| hE-cadherin | GCCCCATCAGGCCTCCGTTT | ACCTTGCCTTCTTTGTCTTTGTTGGA |
| hCD31 | GCTGACCCTTCTGCTCTGTT | TGAGAGGTGGTGCTGACATC |
| hCD45 | AACAGTGGAGAAAGGACACA | TGTGTCCAGAAAGGCAAAGC |
| hαSMA | CCGGGAGAAAATGACTCAAA | GAAGGAATAGCCACGCTCAG |
| hCOL1α1 | GAGGGCCAAGACGAAGACATC | CAGATCACGTCATCGCACAAC |
| mβ-actin | AGGTCATCACTATTGGCAACGA | CCAAGAAGGAAGGCTGGAAAA |
| mE-cadherin | GTCTCCTCATGGCTTTGC | CTTTAGATGCCGCTTCAC |
| mCD31 | CTCCAACAGAGCCAGCAGTA | GACCACTCCAATGACAACCA |
| mCD45 | GAGGTGTCTGATGGTGCAAG | TGTATTCCACTAAAGCCTGATGAA |
| mαSMA | GTCCCAGACATCAGGGAGTAA | TCGGATACTTCAGCGTCAGGA |
| mCOL1α1 | GGAACTTGGGGCAAGACAGT | GAGGGAACCAGATTGGGGTG |
| S4 reovirus | CGCTTTTGAAGGTCGTGTATCA | CTGGCTGTGCTGAGATTGTTTT |

**Supplementary table 1.** Primer sequences used for RT-qPCR.

| **Fibroblast** | **JAM-A** | **CAR** |
| --- | --- | --- |
| SLC052 | 1844 (++) | 1326 (++) |
| SLN052 | 214 (+-) | 726 (+) |
| SLC069 | 1586 (++) | 162 (-) |
| EN002 | 827 (+) | 147 (-) |
| EN004 | 357 (+-) | 657 (+) |
| MC034 | 540 (+) | 94 (-) |
| MN034 | 369 (+-) | 260 (+-) |
| MC057 | 191 (-) | 1014 (++) |
| MN057 | 420 (+-) | 179 (-) |
| MC072 | 344 (+-) | 238 (+-) |
| MC102 | 163 (-) | 312 (+-) |
| MN102 | 108 (-) | 183 (-) |
| DC017 | 305 (+-) | 285 (+-) |
| DN017 | 357 (+-) | 202 (+-) |
| DC027 | 120 (-) | 4106 (++) |
| DC083 | 2752 (++) | 140 (-) |
| DN083 | 298 (+-) | 722 (+) |
| DC140 | 86 (-) | 3412 (++) |
| DN140 | 1751 (++) | 0 (-) |
| DN161 | 861 (+) | 381 (+-) |
| PC011 | 419 (+-) | 404 (+-) |
| PC021 | 175 (-) | 1247 (++) |
| PN021 | 1160 (++) | 225 (+-) |
| PC025 | 28 (-) | 774 (+) |
| PN025 | 58 (-) | 30 (-) |
| PC036 | 51 (-) | 132 (-) |
| PC037 | 39 (-) | 283 (+-) |
| PC050 | 99 (-) | 143 (-) |
| PC087 | 193 (-) | 527 (+) |
| PC109 | 53 (-) | 321 (+-) |
| PC118 | 112 (-) | 179 (-) |
| PC126 | 277 (+-) | 336 (+-) |
| PC129 | 52 (-) | 303 (+-) |
| PC135 | 73 (-) | 128 (-) |

**Supplementary table 2.** Mean Fluorescence Intensity of JAM-A and CAR expression on GI fibroblasts as determined by flow cytometry (++: MFI >1500, +: MFI 500-1500, +/-: MFI 250-500, -: MFI<250).

*Supplementary figure legends*

**Supplementary figure 1.** Human gastrointestinal fibroblast validation. **A** Primary gastrointestinal fibroblasts were cultured for 4 passages and fibroblast identity was subsequently confirmed with RT-qPCR for the expression of Collagen1α1 (Col1a1), alpha smooth muscle actin (aSMA), CD31, CD45 and E-cadherin. **B** Heat map depicts expression of Collagen1α1 (Col1a1), alpha smooth muscle actin (aSMA), CD31, CD45 and E-cadherin.

**Supplementary figure 2.** Ad5-Δ24 efficacy. Cell viability of BxPC3 pancreatic tumor cells 5 days post-infection with Ad5-Δ24.

**Supplementary figure 3.** JAM-A expression of primary pancreatic fibroblasts. FACS analysis of JAM-A expression on JAM-A negative (PN014) and JAM-A positive (PN021) fibroblasts.

**Supplementary figure 4.** Murine pancreatic fibroblast validation. **A** Pancreatic fibroblasts cultured from KPC3 tumors were validated by measuring expression of Collagen1α1 (Col1a1), alpha smooth muscle actin (aSMA), CD31, CD45 and E-cadherin using RT-qPCR. **B** PCR analysis on genomic DNA extracted from KPC3 tumor cells and the different primary fibroblast cultures (KPC3-CAF1, KPC3-CAF3) for expression of the mutant KRAS allele.

**Supplementary figure 5.** Viral titration in pancreatic stellate cells. hPS1 fibroblasts expressing the vector control or full-length JAM-A were infected at different a MOI with either R124 (R) and *jin-3* (J). The Western blot shows sigma(σ)-3 protein expression.

**Supplementary figure 6.** Human organoid-fibroblast co-cultures validation. Protein expression of cytokeratin (organoid) and vimentin (fibroblast) in human organoid-fibroblast co-cultures.

**Supplementary figure 7.** β1-integrin surface expression on hPS1 fibroblasts. Expression of β1-integrin hPS1 fibroblasts expressing the vector control (hPS1_vector), full-length JAM-A (hPS1_fullJAM), or mutant JAM-A that lack the intracytoplasmic tail (hPS1_ΔicJAM) or the terminal PDZ-domain (hPS1_ ΔpdzJAM).

**Supplementary figure 8.** Necroptosis induction in hPS1 fibroblasts. Expression of phosphorylated and total MLKL in hPS1 fibroblasts and the positive control cell line HT29. M=mock, R=R124, J=*jin*-3, T=TBZ.
