# Supplementary figures and images for: Gastrointestinal cancer-associated fibroblasts expressing Junctional Adhesion Molecule-A are amenable to infection by oncolytic reovirus
Source: Cancer Gene Ther. 2022 Jul 22;29(12):1918–29. doi: 10.1038/s41417-022-00507-9 (PMC9750869; doi:10.1038/s41417-022-00507-9)

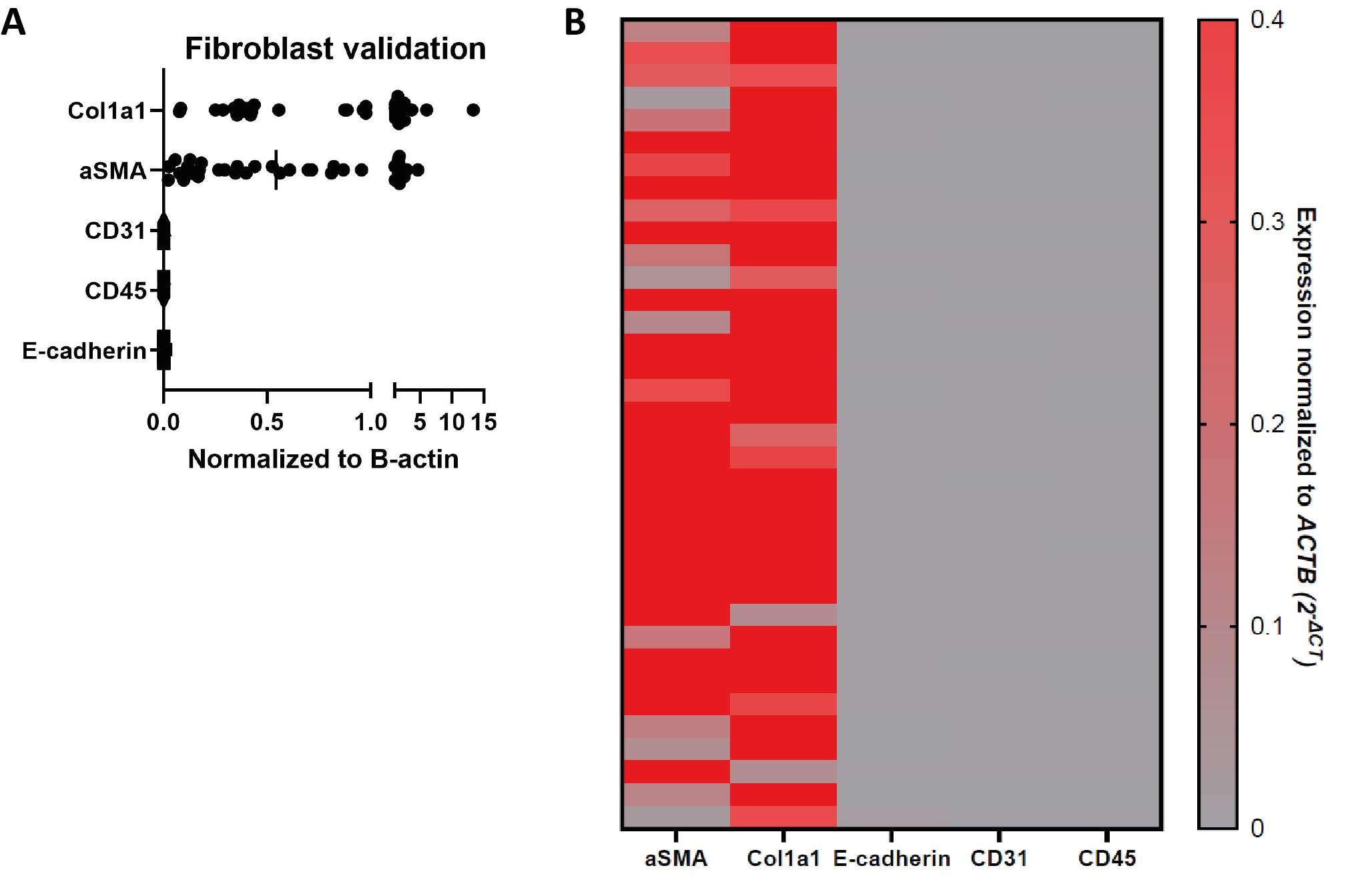

Supplement: Supplementary file 2 — Supplementary figure 1 [file 41417_2022_507_MOESM2_ESM.png]

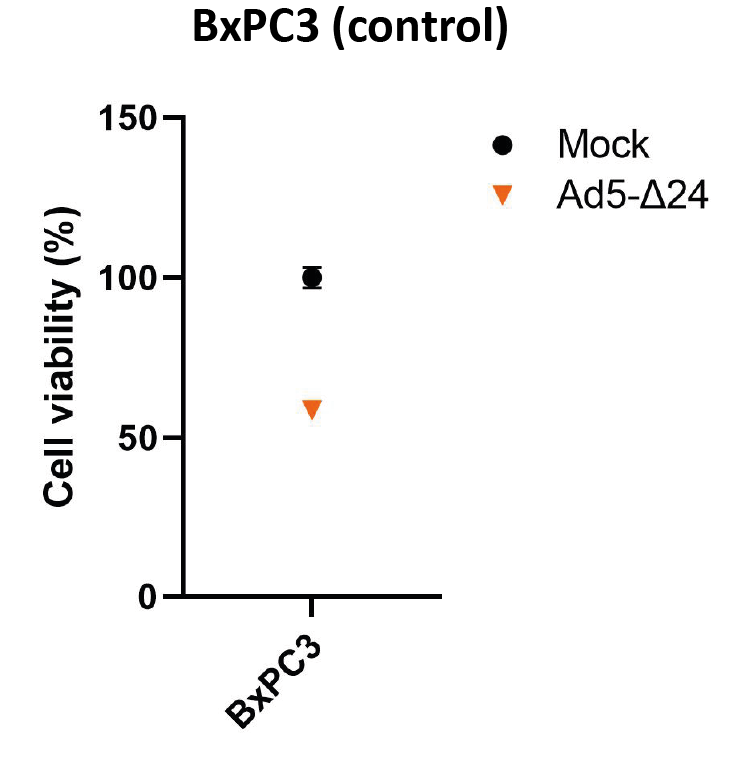

Supplement: Supplementary file 3 — Supplementary figure 2 [file 41417_2022_507_MOESM3_ESM.png]

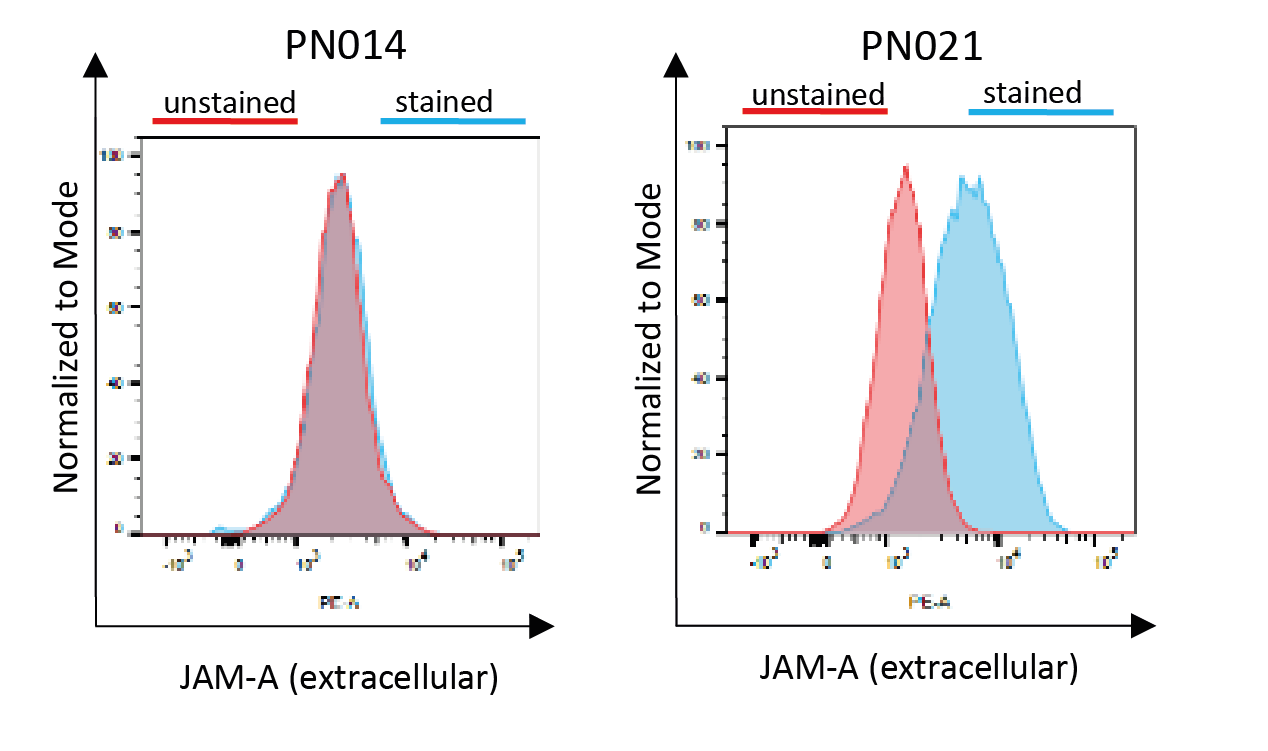

Supplement: Supplementary file 4 — Supplementary figure 3 [file 41417_2022_507_MOESM4_ESM.png]

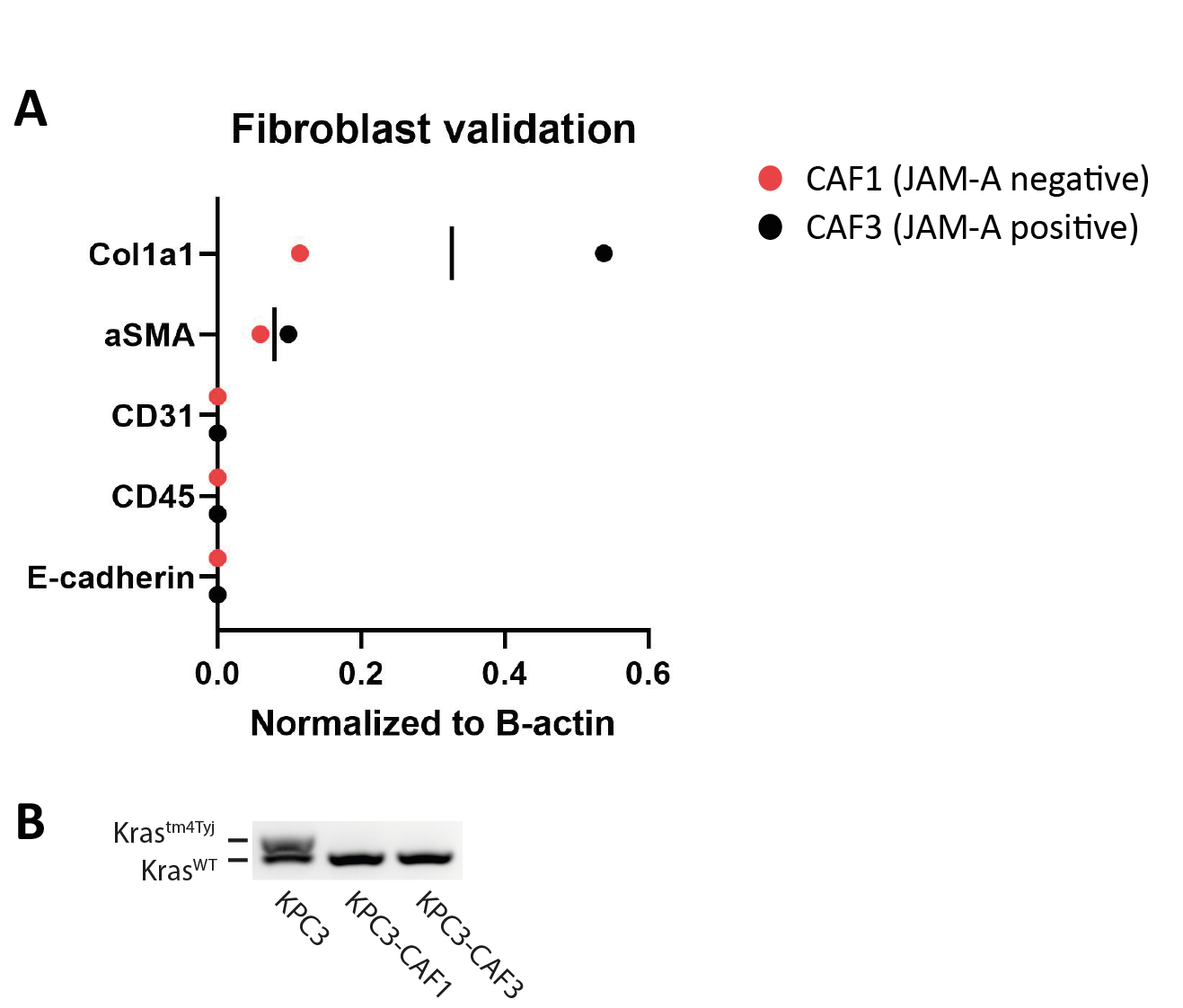

Supplement: Supplementary file 5 — Supplementary figure 4 [file 41417_2022_507_MOESM5_ESM.png]

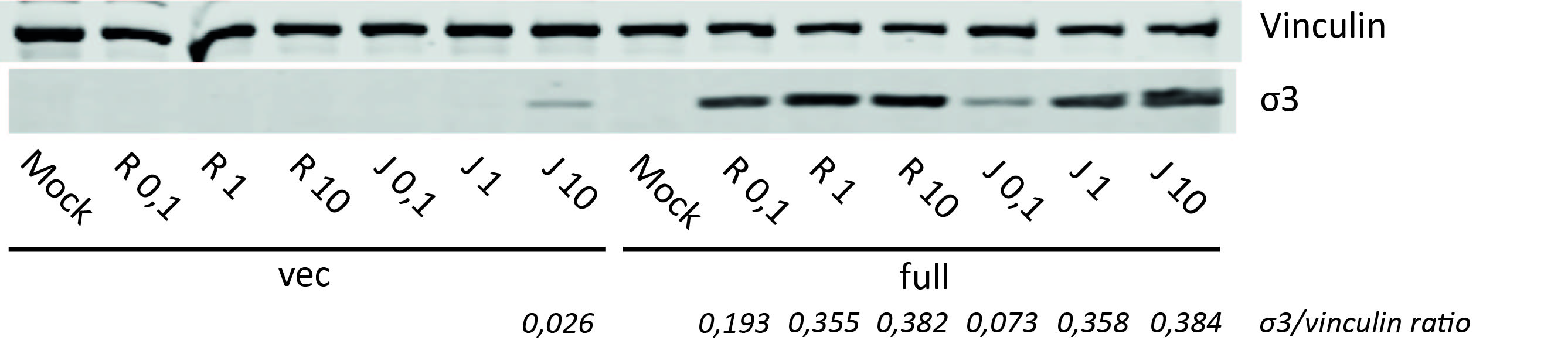

Supplement: Supplementary file 6 — Supplementary figure 5 [file 41417_2022_507_MOESM6_ESM.jpg]

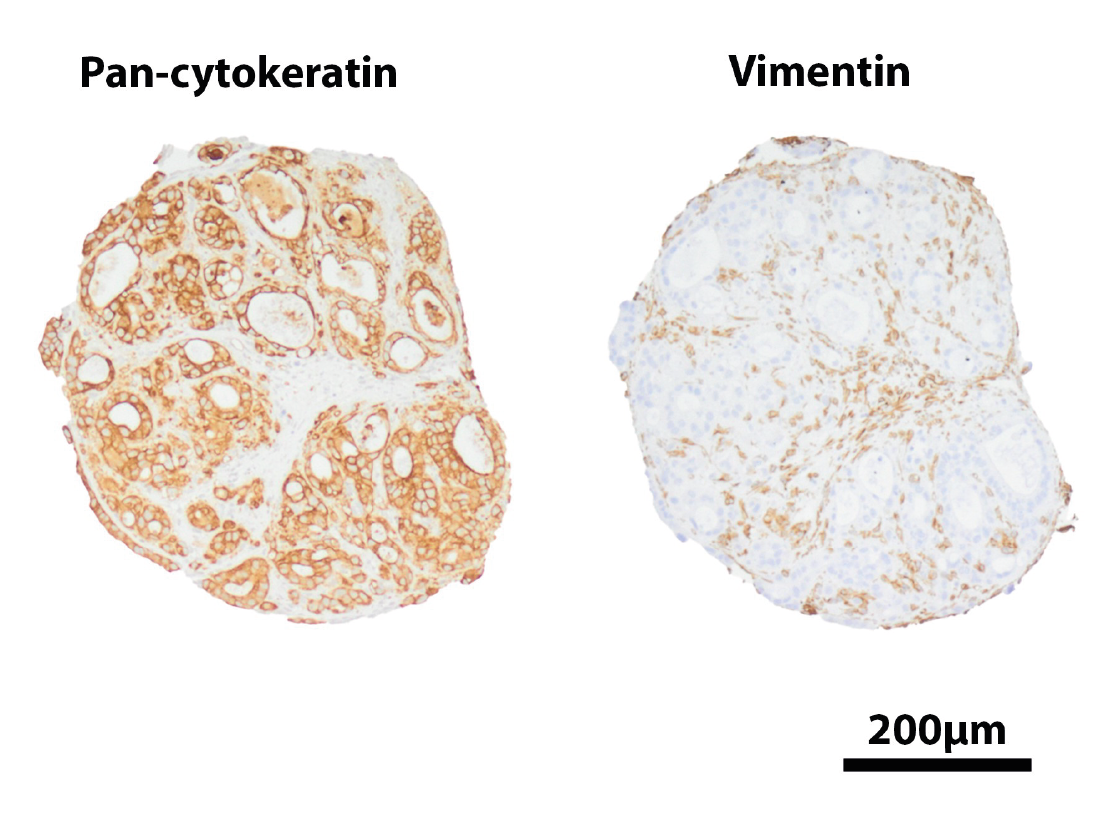

Supplement: Supplementary file 7 — Supplementary figure 6 [file 41417_2022_507_MOESM7_ESM.png]

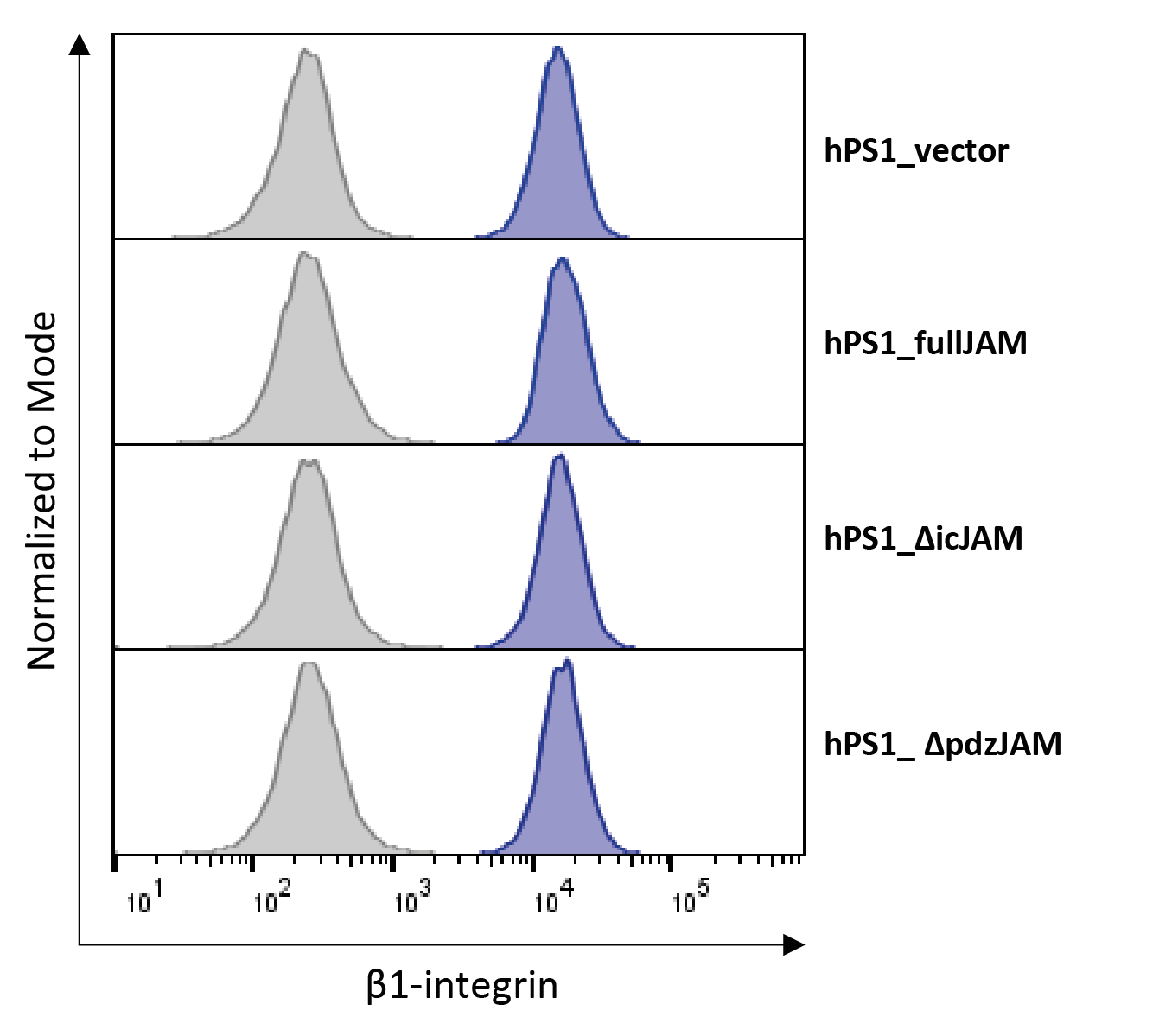

Supplement: Supplementary file 8 — Supplementary figure 7 [file 41417_2022_507_MOESM8_ESM.png]

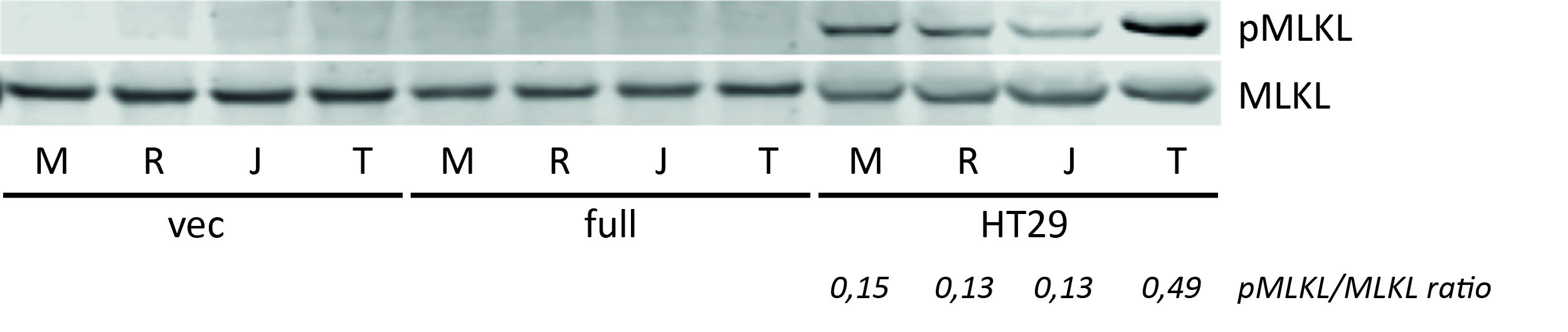

Supplement: Supplementary file 9 — Supplementary figure 8 [file 41417_2022_507_MOESM9_ESM.jpg]
